# Lithium promotes neural precursor cell proliferation: evidence for the involvement of the non-canonical GSK-3β-NF-AT signaling

**DOI:** 10.1186/2045-3701-1-18

**Published:** 2011-05-03

**Authors:** Zhaoxia Qu, Dongming Sun, Wise Young

**Affiliations:** 1 Department of Cell Biology and Neuroscience, W. M. Keck Center for Collaborative Neuroscience, Rutgers, The State University of New Jersey, Piscataway, NJ 08854, USA; 2 University of Pittsburgh Cancer Institute, Department of Microbiology and Molecular Genetics, University of Pittsburgh School of Medicine, Pittsburgh, PA 15213, USA

## Abstract

Lithium, a drug that has long been used to treat bipolar disorder and some other human pathogenesis, has recently been shown to stimulate neural precursor growth. However, the involved mechanism is not clear. Here, we show that lithium induces proliferation but not survival of neural precursor cells. Mechanistic studies suggest that the effect of lithium mainly involved activation of the transcription factor NF-AT and specific induction of a subset of proliferation-related genes. While NF-AT inactivation by specific inhibition of its upstream activator calcineurin antagonized the effect of lithium on the proliferation of neural precursor cells, specific inhibition of the NF-AT inhibitor GSK-3β, similar to lithium treatment, promoted neural precursor cell proliferation. One important function of lithium appeared to increase inhibitory phosphorylation of GSK-3β, leading to GSK-3β suppression and subsequent NF-AT activation. Moreover, lithium-induced proliferation of neural precursor cells was independent of its role in inositol depletion. These findings not only provide mechanistic insights into the clinical effects of lithium, but also suggest an alternative therapeutic strategy for bipolar disorder and other neural diseases by targeting the non-canonical GSK-3β-NF-AT signaling.

## Introduction

Lithium is a monovalent cation belonging to the group of alkali metals. It has been the reference standard medication for acute and prophylactic treatment of bipolar disorder/manic depressive illness, a brain disorder in which normal moods alternate with both depression and mania, which is recognized by the World Health Organization as a leading debilitating neuropsychiatric disorder that affects about 1.3% of both sexes globally [1]. Recent animal studies suggest a beneficial effect of lithium on other central nervous system (CNS) diseases, such as brain ischemia, spinal cord injury, Alzheimer's disease and Huntington's disease [2].

Currently, two major targets of lithium are suggested responsible for the actions of lithium on bipolar disorder and other CNS diseases: inositol depletion and glycogen synthase kinase 3β (GSK-3β) inhibition. Lithium inhibits inositol polyphosphate 1-phosphatase (IPPase) and inositol monophosphate phosphatase (IMPase), two enzymes critical for the recycling and de novo synthesis of inositol, thereby leading to inositol depletion [3]. Lithium may also reduce inositol uptake from outside of cells by down-regulating expression of inositol transporter gene such as sodium-myo-inositol transporter 1 (SMIT1) [4]. In support of the concept that inositol depletion may be the way that lithium works in bipolar disorder and other CNS diseases, inositol depletion mice due to the *smit1 *gene homozygous deletion behave similarly to lithium-treated animals [5]. However, much higher inositol depletion is required for achievement of the behavioral effects in mice than that achieved by lithium administration [6], suggesting that the inositol depletion role of lithium is not responsible for all its actions.

More and more studies suggest that inhibition of GSK-3β may be a more relevant target for the pathophysiology of bipolar disorder and the therapeutic action of lithium [7]. For example, loss of GSK-3 function in Xenopus and Dictyostelium results in developmental abnormalities that are phenocopied by lithium treatment [8,9]. More importantly, mice with heterozygous loss of GSK-3β genotype exhibit behavioral and molecular changes similar to those induced by lithium treatment [10], and transgenic mice overexpressing GSK-3β show hyperactivity resembling that observed in the manic phase of bipolar disorders [11]. In agreement with the *in vivo *role of GSK-3β in inhibition of neural precursor cell proliferation [12], GSK-3β inhibition is also involved in lithium-mediated proliferation of human NT2 neural-like precursor cells and proliferation recovery of dexamethasone-treated adult rat dentate gyrus-derived neural precursor cells (ADP) [13,14].

GSK-3β is a serine/threonine kinase that has diverse functions in various cellular activities in many cell types, including glycogen synthesis, cell survival and cell division [15]. Unlike most protein kinases, GSK-3β is constitutively active and its activity is down-regulated by upstream signals through inhibitory phosphorylation. The most important and well-known target of GSK-3β is the β-catenin transcriptional coactivator. Active GSK-3β can directly phosphorylate β-catenin, resulting in ubiquitination-medaited proteasomal degradation of β-catenin. The NF-AT transcription factor has been found to be another target of GSK-3β, at least in T cells and neurons [16,17]. Different from the β-catenin phosphorylation, NF-AT phosphorylation mediated by GSK-3β promotes its export from the nucleus, therefore terminating NF-AT-dependent transcription [18]. The NF-AT activation is delicately counterbalanced by GSK-3β and Ca^2+^-calcineurin. GSK-3β phosphorylates NF-AT, leading to its nuclear export and transcriptional inactivation, while Ca^2+^-calcineurin dephosphorylates NF-AT, leading to its nuclear import and transcriptional activation.

Currently, the two models have not been well reconciled yet. Part of the reasons might be due to that the outcome of lithium administration may be cell type dependent. In the present study, we showed that lithium promoted proliferation but not survival of neural precursor cells. Consistently, we found that lithium specifically induced expression of a subset of cell proliferation-related genes in these cells. Whereas addition of inositol had no effect on lithium-induced neural precursor cell growth, inhibition of GSK-3β showed an effect similar to lithium. On the other hand, inhibition of calcineurin/NF-AT antagonized the effect of lithium on neural precursor cell proliferation. Although lithium administration was able to increase inhibitory phosphorylation of GSK-3β, it failed to stabilize β-catenin. These studies suggested that targeting GSK-3β for NF-AT activation is the main mechanism for lithium-induced neural precursor cell proliferation.

## Results

### Lithium increases numbers of neural precursor cells in culture

**Figure 1:**
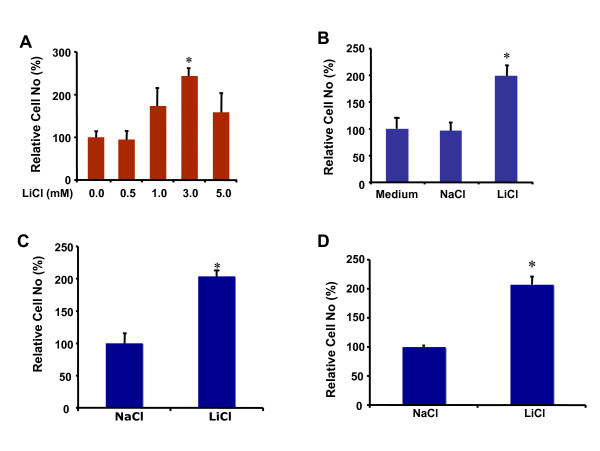
**Lithium induced cell number increase in neural precursor cultures**. (A) Lithium chloride dose dependently stimulated cell number increase in RG3.6 cell cultures. Cell count analysis was performed on cultures of RG3.6 cells treated with the indicated doses of LiCl for 7 days. *: Bonferroni/Dunn, P < 0.002 compared to no LiCl control. (B) Lithium chloride but not sodium chloride induced cell number increase in RG3.6 cell cultures. Cell count analysis was performed on cultures of RG3.6 cells treated with control medium, 3 mM NaCl or 3 mM LiCl for 5 days. *: paired t test, P < 0.01 compared to 3 mM NaCl or control medium treatment. (C) Lithium stimulated cell number increase in rat primary neural precursor cell cultures. Cell count analysis was performed on cultures of rat primary neural precursor cells treated with 3 mM NaCl or 3 mM LiCl for 5 days. *: Bonferroni/Dunn, P < 0.002. (D) Lithium stimulated cell number increase in mouse primary neural precursor cell cultures. Cell count analysis was performed on cultures of mouse primary neural precursor cells treated with 3 mM NaCl or 3 mM LiCl for 5 days. *: Bonferroni/Dunn, P < 0.002. The data represent mean ± standard error (n = 6 for each condition).

Given its clinical benefit on manic depressive illness and its potential application for other central nervous system diseases, we investigated the effects of lithium on growth of neural precursor cell line RG3.6 cells as well as primary neural precursor cells isolated from neonatal mouse and rat. As shown in Figure 1A, lithium chloride (LiCl) treatment resulted in a dose-dependent cell number increase in RG3.6 cell cultures with the maximal effect at 3 mM. In contrast, 3 mM sodium chloride (NaCl) treatment had no obvious effect on RG3.6 cell numbers (Figure 1B), indicating that the lithium, but not the chloride, in LiCl was responsible for the cell number increase. Similarly, lithium significantly induced cell number increase in mouse and rat primary neural precursor cell cultures (Figures 1C and 1D). These results suggested a general role of lithium in cell number increase of various neural precursor cells.

### Lithium has no significant effect on the survival of neural precursor cells

**Figure 2:**
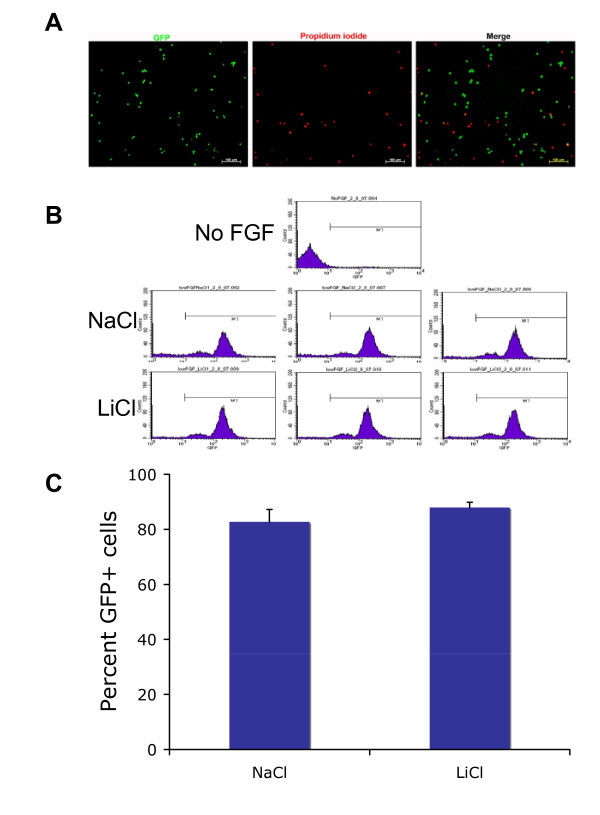
**Lithium had no significant effect on percentage of GFP expressing live cells in RG3.6 cell cultures**. (A) GFP-expressing cells are negative for propidium iodide (PI) staining. RG3.6 neurospheres were dissociated by trypsinization. The dissociated cells were stained with PI, a red fluorescent nuclear dye that specifically stain dead cells. The green GFP signal and the red PI staining were visualized and photographed using fluorescent microscope. (B) RG3.6 cells treated with NaCl or LiCl had similar GFP histograms. Flow cytometry analysis was performed on RG3.6 cell cultures treated with 3 mM NaCl or 3 mM LiCl for 3 days. Cells grown in non-FGF2 containing medium for 6 days were used as a negative control to gate GFP signal, since most cells grown in this condition were dead. (C) Quantification of the data shown in B. Lithium treatment had no significant effect on the percentage of GFP-expressing live cells in RG3.6 cell cultures.

To investigate the mechanism by which lithium induces cell number increase of neural precursor cells, we analyzed the effect of lithium on survival of RG3.6 cells. The RG3.6 cell line was originally derived from GFP transgenic rat [19]. When these cells die, they lose their GFP signal, as revealed by the mutual exclusiveness of GFP signal with the red fluorescent signal due to propidium iodide staining, a hallmark of dead cells (Figure 2A). Therefore, GFP expression in these cells provides a convenient marker for survival cells. Accordingly, we performed flow cytometry assay to compare the percentage of GFP-expressing live cells in RG3.6 cell cultures treated with 3 mM LiCl or control NaCl. Surprisingly, LiCl treatment failed to increase the ratio of survival cells, compared to NaCl treatment (Figures 2B and 2C). Nevertheless, this result indicated that lithium has no significant effect on neural precursor cell survival, excluding the possible role of cell survival in lithium-induced cell number increase.

### Lithium promotes proliferation of neural precursor cells

**Figure 3:**
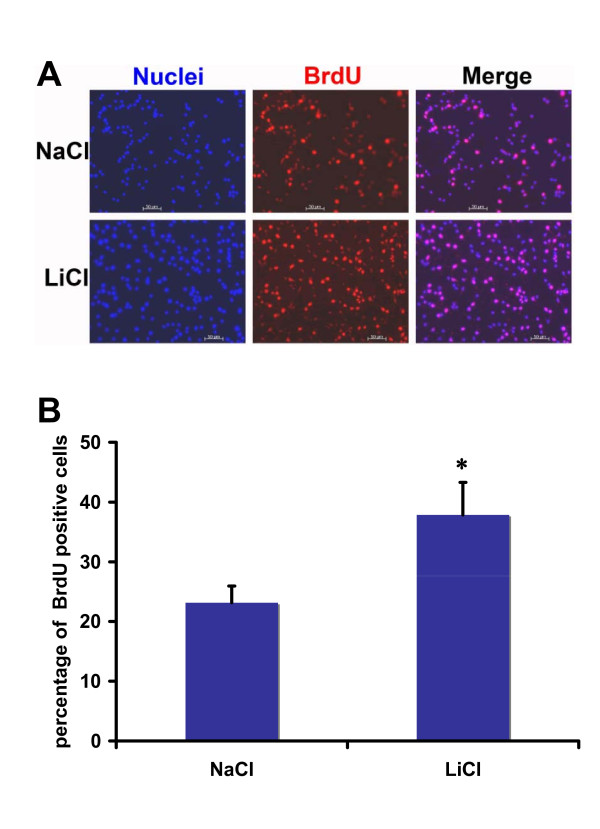
**Lithium significantly increased percentage of BrdU^+ ^cells in RG3.6 cell cultures**. RG3.6 cells were grown in culture medium containing 3 mM LiCl or control NaCl on laminin-coated coverslips for 3 days followed by 4 hours of BrdU labeling and subsequent BrdU immuno-staining. The data represent mean ± standard error. *: Bonferroni/Dunn, P < 0.0001, n = 8 for each condition.

The results above further suggest that lithium may promote proliferation of neural precursor cells, which accounts for the lithium-induced cell number increase. To verify this, we measured cell proliferation by performing bromodeoxyuridine (BrdU) incorporation assay to label dividing cells. In the NaCl control group, about 23% neural precursor cells were BrdU positive after 4 hours of BrdU incubation (Figure 3A and 3B). However, the ratio of the BrdU positive cells was increased to 38% in the group treated with LiCl. Together, these results suggest that lithium stimulates proliferation, but not survival, of neural precursor cells.

### Lithium induces a subset of cell proliferation-related genes

**Figure 4:**
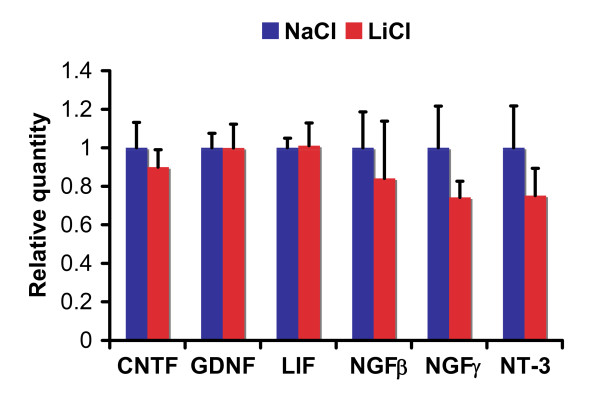
**Lithium had no significant effect on RNA expression of neurotrophic factors CNTF, GDNF, LIF, NGFβ, NGFγ and NT-3 in rat primary neural precursor cells**. Rat primary neural precursor cells were treated with 3 mM LiCl or control NaCl for 3 days. Then the cells were lysed for RNA extraction and subsequent quantitative real time PCR analysis. RNA levels of neurotrophic factors were normalized to peptidylprolyl isomerase A (Ppia). The data represent mean ± standard error. *: Bonferroni/Dunn, P < 0.05, n = 3 for each condition.

To further confirm our finding above, we performed microarray analysis to compare gene expression profiles in RG3.6 neural precursor cells treated with LiCl or control NaCl. Consistent with our proliferation assay, many cell proliferation-related genes were significantly up-regulated upon lithium treatment (Additional file 1). Notably, none of the known neurotrophic factors, such as NGFβ, NGFγ, NT-3, BDNF, CNTF, GDNF and LIF, were induced by lithium. Our real-time PCR using primary neural precursor cells further demonstrated that lithium had no significant effect on RNA expression of NGFβ, NGFγ, NT-3, CNTF, GDNF and LIF (Figure 4). Thus, lithium-induced increase in neural precursor cell numbers is mainly due to lithium-induced stimulation of cell proliferation rather than improvement of cell survival.

### Myo-inositol has no significant effect on lithium-induced proliferation of neural precursor cells

**Figure 5:**
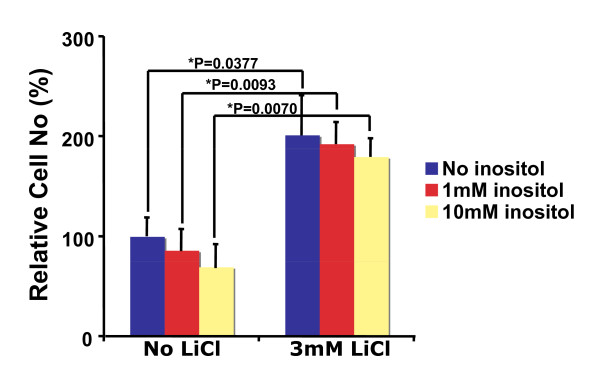
**Inositol had no significant effect on lithium-induced cell number increase in RG3.6 cell cultures**. Cell count analysis was performed on RG3.6 cells cultured for 5 days in medium with or without 3 mM LiCl, and with or without 1 mM or 10 mM myo-inositol (n = 6 for each condition). The data represent mean ± standard error, and significance was determined with Fisher's PLSD post hoc test following analysis of variance (ANOVA).

Since inositol depletion contributes to lithium action in the central nervous system [5], we examined whether addition of extracellular inositol blocks lithium-induced neural precursor cell growth. As shown in Figure 5, up to 10 mM of myo-inositol did not significantly affect growth of RG3.6 cells during 5 days incubation. More importantly, the same concentration of myo-inositol failed to inhibit lithium-induced cell number increase. This result suggests that inositol depletion is not the mechanism by which lithium promotes neural precursor cell proliferation.

### Lithium-induced neural precursor cell proliferation involves GSK-3β suppression

**Figure 6:**
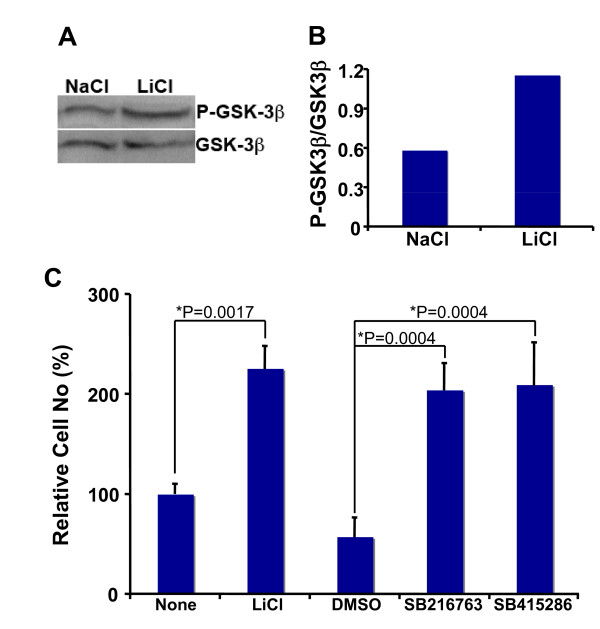
**Lithium inhibited GSK-3β in RG3.6 cells, and other GSK3β inhibitors mimicked lithium's effect on RG3.6 cell growth**. (A) RG3.6 cells were grown in culture medium containing 3 mM LiCl or 3 mM control NaCl for 3 days, followed by western blotting analysis on P-GSK-3β (Ser9) and GSK-3β expression. (B) The blotting results in A were analyzed using ImageJ software (http://rsb.info.nih.gov/ij/, 1997-2007), and the ratio of P-GSK-3β was plotted. (C) Other GSK-3β inhibitors, like lithium, also increased cell numbers in RG3.6 cell cultures. Cell count analysis was performed on RG3.6 cells grown for 6 days in culture medium without or with 3 mM LiCl, 0.1% DMSO, 5 μM SB216763, or 25 μM SB415286 (n = 6 for each condition). DMSO was used as the vehicle control for SB216763 and SB415286, since these two drugs were dissolved in DMSO. The data represent mean ± standard error, and significance was determined with Bonferroni/Dunn post hoc analysis following ANOVA.

Lithium has been shown to inhibit glycogen synthase kinase 3β (GSK-3β) activity in many types of cells [8,20-24]. Thus, we examined whether GSK-3β inhibition is involved in lithium-induced proliferation of neural precursor cells. First, we examined whether lithium inhibits GSK-3β in neural precursor cells by detecting phosphorylation levels of GSK-3β at serine 9 in the presence of LiCl or NaCl. Phosphorylation of this serine is one important mechanism leading to GSK-3β inactivation. Consistent with its inhibitory role in other cell types, lithium also significantly increased phosphorylation of GSK-3β at serine 9 in RG3.6 cells (Figures 6A and 6B). This result suggests that lithium suppresses GSK-3β activity in neural precursor cells.

Next, we tested the potential effects of other GSK-3β inhibitors on the proliferation of neural precursor cells. If lithium-mediated suppression of GSK-3β plays a role in neural precursor cell proliferation, other GSK-3β inhibitors should have the effects similar to lithium on RG3.6 cell growth. For this purpose, we utilized two potent and specific GSK-3 inhibitors, SB216763 and SB415286. Indeed, both SB216763 and SB415286 treatments resulted in significant cell number increase, just like lithium treatment (Figure 6C). Collectively, these results suggest that inhibition of GSK-3β contributes to lithium-induced neural precursor cell proliferation.

### Calcineurin/NF-AT inhibitor cyclosporin A antagonizes lithium-induced neural precursor cell proliferation

**Figure 7:**
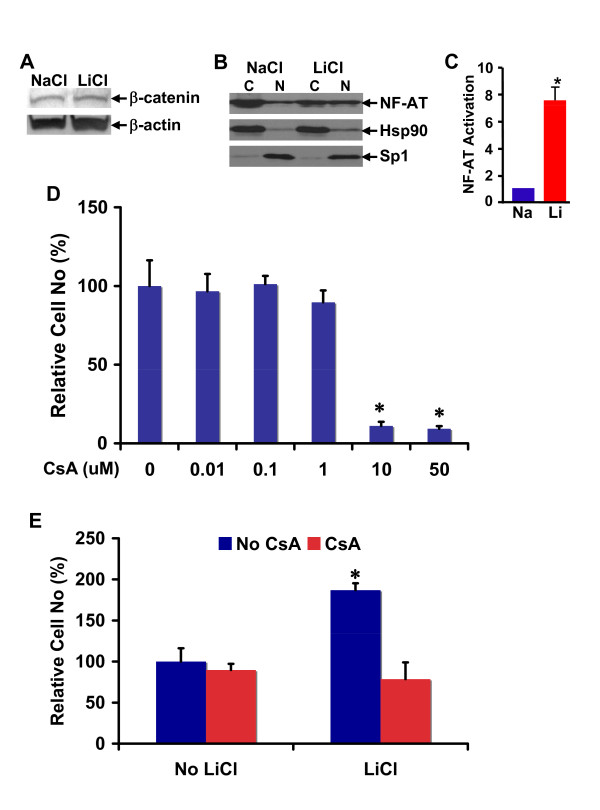
**Calcineurin/NFAT inhibitor cyclosporin A antagonized lithium-induced cell number increase in RG3.6 cell cultures**. (A) Lithium did not significantly change β-catenin expression in RG3.6 cells. RG3.6 cells were grown for 3 days in culture medium containing 3 mM LiCl or 3 mM control NaCl. The cells were then lysed in RIPA for western blotting analysis on β-catenin and β-actin expression. (B) Lithium increased nuclear expression of NF-AT. Aliquot of cells from A were used for cytoplamic and nuclear fractionation, followed by western blotting assays for the subcellular expressions of NF-AT. (C) Lithium stimulated transcriptional activation of NF-AT. RG3.6 cells treated with LiCl or NaCl were used for gene reporter assays. The luciferase activity is presented as fold induction relative to that of NaCl-treated cells. *: paired t test, P < 0.01. (D) Effect of different doses of cyclosporin A (CsA) on RG3.6 cell growth. Cell count analysis was performed on RG3.6 cells grown for 5 days in culture medium containing various doses of CsA (0 (Control), 0.01 μM, 0.1 μM, 1 μM, 10 μM, 50 μM, n = 6 for each dose). *: paired t test, P < 0.005 compared to control, 0.01 μM, 0.1 μM, or 1 μM CsA condition. (E) Cyclosporin A antagonized lithium-induced RG3.6 cell growth. Cell count analysis was performed on RG3.6 cells grown for 5 days in culture medium with or without 3 mM LiCl, and with or without 1 μM CsA (n = 6 for each condition). The data represent mean ± standard error. *: paired t test, P < 0.02 compared to No CsA + No LiCl, or CsA + No LiCl condition.

To find GSK-3β downstream signaling involved in lithium-induced neural precursor cell proliferation, we analyzed β-catenin and NF-AT: two most important targets of GSK-3β. GSK-3β inhibits both β-catenin and NF-AT by directly phosphorylating them. While GSK-3β-mediated NF-AT phosphorylation results in its retention in the cytoplasm, β-catenin phosphorylation by GSK-3β leads to its proteasomal degradation. Thus, we examined the effect of lithium treatment on β-catenin protein stability and NF-AT subcellular expression in RG3.6 neural precursor cells. As revealed by our immunoblotting assays, lithium treatment had no obvious effect on β-catenin protein expression (Figure 7A) but significantly increased nuclear translocation of NF-AT (Figure 7B). Consistently, the lithium treatment also led to the transcriptional activation of NF-AT (Figure 7C). This result suggests that NF-AT, but not β-catenin, may be involved in lithium-induced proliferation of neural precursor cells.

GSK-3β-mediated inhibition of NF-AT is counterbalanced by Ca^2+^-calcineurin. Ca^2+^-calcineurin dephosphorylates and thereby activates NF-AT. To investigate the role of NF-AT in lithium-induced proliferation of neural precursor cells, we took advantage of cyclosporin A (CsA), a specific inhibitor of the NF-AT activator calcineurin. If NF-AT activation is involved in lithium-induced proliferation of neural precursor cells, CsA should suppress the effect of lithium. As shown in Figure 7D, CsA alone, at doses of up to 1 μM, did not significantly affect RG3.6 cell growth, although at doses of 10 μM or above it significantly reduced RG3.6 cell numbers. More interestingly, CsA, even at the low dose (1 μM) that does not affect cell growth, completely abolished lithium-induced RG3.6 cell number increase (Figure 7E). Together, these results suggest that lithium-induced proliferation of neural precursor cells involves GSK-3β suppression and subsequent NF-AT activation.

## Discussion

Lithium is a standard medication for bipolar disorder. Recent in vitro and animal studies suggest a great potential of lithium in the treatment of other central nervous system (CNS) diseases, although the involved mechanisms remain elusive. Here, we have shown that lithium promotes growth of neural precursor cells. Lithium stimulates neural precursor cell proliferation but not their survival. Our mechanistic studies further indicate that lithium-induced neural precursor cell proliferation involves the non-canonical GSK-3β-NF-AT signaling, but not inositol depletion or β-catenin, the canonical downstream signaling of GSK-3β.

Neurotrophic factors are well known for their roles in neuron growth. Interestingly, it has been reported that lithium induces expression of neurotrophic factors in specific regions of rodent brain [25-30], although the resource cells for the neurotrophic factors and the exact functions of their induction await investigation. One of the potential resources is neurons, because lithium increases neurotrophic factor expression in cultured cortical neurons, which is essential for neuroprotection under certain stress conditions [31]. However, lithium failed to up-regulate neurotrophic factors in neural precursor cells (Figure 4 and Additional file 1), suggesting that neural precursor cells are not additional resources for the survival factors. Instead, lithium significantly enhanced expression of numerous proliferation-related genes (Additional file 1). In results consistent with the gene induction profile, lithium only promoted proliferation but not survival of neural precursor cells (Figures 1, 2 and 3). These data were also consistent with the fact that neurotrophic factors are not required for growth of neural precursor cells, at least in vitro. These studies together thus suggest that the effects of lithium are cell type dependent.

Two different hypotheses have been proposed to explain the lithium's mood-stabilizing properties: inositol depletion and GSK-3 inhibition. However, it remains obscure how these two hypotheses are integrated. Although increasing evidence has suggested a relatively more dominant role of GSK-3 inhibition in the actions of lithium [7], it seems highly plausible that inositol depletion and inhibition of GSK-3 both contribute to the therapeutic actions of lithium; because either inositol depletion or GSK-3β deficient animals exhibit phenotypes the same as, although similar to, those induced by lithium treatment [5,10]. Given the important role of inositol in various signaling pathways including the GSK-3 pathway and the essential role of GSK-3 in the optimal de novo synthesis of inositol [32], the two different mechanisms possibly also together with other mechanisms collaboratively fine-tune animal's responses to lithium.

It is likely that the relative importance of each mechanism is also context dependent. For example, insoitol depletion and GSK-3β inhibition are responsible for lithium-induced growth cone spread in dorsal root ganglion sensory neurons and neuronal polarity of hippocampal neurons, respectively [26-35]. In the hippocampal neuron polarity, collapsin response mediator protein-2 (CRMP-2), a factor critical for specifying axon/dendrite fate by promoting neurite elongation via microtubule assembly, is the major target of GSK-3β [33]. Our studies here suggest that lithium stimulates proliferation but not survival of neural precursor cells (Figures 1, 2 and 3). Interestingly, inhibition of GSK-3β, but not inositol depletion, is the mechanism accounting for lithium-induced cell proliferation, because lithium treatment led to GSK-3β inhibition and inhibition of GSK-3β by other GSK-3 inhibitors showed an effect similar to lithium (Figure 6). On the other hand, addition of myo-inositol had no effect on lithium-induced neural precursor cell proliferation (Figure 5). We have further found that NF-AT is the major downstream signaling molecule of GSK-3β in lithium-induced neural precursor cell proliferation, because inhibition of NF-AT reversed the effect of lithium (Figure 7). The effect on NF-AT seems highly specific, as lithium-mediated inhibition of GSK-3β failed to stabilize β-catenin in the same cells. Consistent with our studies, lithium also stimulates proliferation of human NT2 neural precursor cells but cannot stabilize β-catenin [16]. Paradoxically, lithium recovers, rather than directly increases, the proliferation of ADP rat neural precursor cells from dexamethasone-mediated inhibition [17]. The difference could be due to the different sources of the neural precursor cells used. NT2 and RG3.6 cell lines were embryonic as well as primary neural precursor cells we used in this study were from neonatal animals. On the other hand, ADP cells were from adult rat. Nevertheless, these studies suggest that lithium utilizes different mechanisms to achieve the same or different effect on different cells. These effects are further integrated, which will decide the final outcome of lithium administration. Thus, understanding when and where each mechanism is most effective will help to fine-tune therapeutic strategies to promote or constrain specific arms of lithium.

## Materials and methods

### Antibodies and reagents

Anti-phospho-GSK-3β (Ser9) and anti-GSK-3β antibodies were purchased from Cell Signaling. Anti-β-catenin antibody was from BD Biosciences. Anti-NF-ATc antibody was from Santa Cruz Biotechnology. Anti-bromodeoxyuridine (BrdU) antibody was from DakoCytomation. Fluorescent dye-conjugated secondary antibodies and 4', 6-Diamidino-2-phenylindole (DAPI) were from Molecular Probes. Horse radish peroxidase-linked anti-rabbit IgG was from Amersham Biosciences. BrdU and myo-inositol were from Sigma-Aldrich. GSK-3β inhibitors SB216763 and SB415286 were from Tocris Cookson, Inc. Calcineurin inhibitor cyclosporin A was from Sandoz Pharmaceuticals. EGF and FGF2 were from BD Biosciences.

### Primary neural precursor cell culture

Olfactory bulbs and subventricular zone tissue samples dissected from neonatal animal brains were suspended in 0.05% trypsin (Sigma-Aldrich), minced by sequentially passing through 18-, 21- and 25-gauge needles, and then incubated at 37°C for 5 minutes to dissociate the cells. After adding an equal volume of trypsin inhibitor (0.25 mg/ml, Sigma-Aldrich), the cells were further dissociated by pipetting up and down. After washed twice with DMEM/F12 medium (Invitrogen), the cell samples were re-suspended in growth medium, i.e. DMEM/F12 supplemented with 25 mM glucose (Sigma-Aldrich), 1X B27 (Invitrogen), 10 ng/ml FGF2 (BD Biosciences) and 10 ng/ml EGF (BD Biosciences), filtered through a 70 μm nylon mesh, and cultured at 37°C in a humidified atmosphere of 95% air + 5% CO_2_. After 24 hrs of culturing, the cells were washed three times with DMEM/F12 and pipetted up and down for dissociation at each wash. The cells were then re-suspended in new growth medium, filtered through a 40 μm nylon mesh and incubated at 37°C in a humidified atmosphere of 95% air + 5% CO_2_. One-tenth of the growth medium was replenished with DMEM/F12 supplemented with 100 ng/ml EGF, 100 ng/ml FGF2, and 1X B27 every other day. For treatment, passage 3 neural precursor cells were cultured in DMEM/F12 supplemented with 25 mM glucose (Sigma-Aldrich), 1X B27, 0.2 ng/ml EGF, 0.2 ng/ml FGF2, in the presence of 3 mM lithium chloride (LiCl) or 3 mM control sodium chloride (NaCl) before the cultures were stopped for analysis.

### RG3.6 cell culture

Rat neural precursor cell line RG3.6 was kindly provided by Dr. Martin Grumet [19]. After passage, the RG3.6 cells were grown in culture medium, i.e. DMEM/F12 supplemented with 25 mM glucose, 1X B27, 0.2 ng/ml FGF2, and 2 μg/ml heparin (Sigma-Aldrich), in the absence or presence of various doses of LiCl or 3 mM NaCl before the cultures were stopped for analysis.

### Cell treatment and Cell count analysis

Cells were cultured in 96-well plates in growth medium containing the indicated concentration of NaCl or LiCl in the presence or absence of the indicated concentrations of inositol, CsA, SB216763, SB415286, or mock DMSO for the indicated time points as described [4,14,17]. Quantification of cell number was done using the Cyquant cell proliferation assay kit (Invitrogen) according to the manufacturer's protocol.

### BrdU labeling and immunocytochemistry

RG3.6 neural precursor cells were grown for 3 days on laminin (Invitrogen, 20 μg/ml)-coated coverslips in culture medium containing 3 mM LiCl or 3 mM control NaCl. Then the cells were labeled with BrdU (10 μM) for 4 hours, and then fixed with 4% para-formaldehyde at room temperature for 15 minutes. The fixed cells were treated with 2 M HCl for 30 minutes at room temperature, followed by 3 times wash with borate buffer (0.1 M, pH 8.5). Normal goat serum (NGS, Invitrogen, 2% in PBS-T, i.e. 0.01 M phosphate buffered saline with 0.05% Tween 20) was applied for 30 minutes to block non-specific binding of antibodies. Mouse monoclonal anti-BrdU antibody (DakoCytomation, 1:100 in PBS-T/2% NGS), goat-anti-mouse AlexaFluor 546 (Molecular Probes, 1:500 in PBS-T/2% NGS), and DAPI (4',6-Diamidino-2-phenylindole, Molecular Probes, 1:1000 in PBS-T/2% NGS) nuclear dye were sequentially applied at room temperature for 30, 30 and 10 minutes, respectively, with PBS-T washing for 3 times after each application. After mounting the coverslips onto slides, the fluorescent staining was visualized using fluorescent microscope.

### Flow cytometry

RG3.6 neurospheres formed after grown in culture medium containing 3 mM NaCl or 3 mM LiCl for 3 days were dissociated by treatment with trypsin-EDTA (0.25% trypsin, 1 mM EDT•4Na, Invitrogen) for 3 to 5 minutes at room temperature, and pipetting up and down after adding an equal volume of 0.25 mg/ml trypsin inhibitor (Sigma-Aldrich). The cells were washed twice with 1 × PBS (pH 7.4). Flow cytometry was performed to analyze the GFP signals in the samples using a flow cytometer as described previously [36].

### Subcellular Fractionation and Western blotting

RG3.6 cells were grown in culture medium containing 3 mM NaCl or 3 mM LiCl for 3 days. Cells were lysed in radioimmuoprecipitation assay buffer (RIPA buffer) [50 mM Tris-HCl pH7.4, 150 mM NaCl, 1 mM EDTA, 0.25% Na-deoxycholate, 1% NP-40, 1 mM dithiothreitol (DTT), 1 mM phenylmethylsulfonyl fluoride (PMSF)] supplemented with a protease inhibitor mixture for whole lystes [37]. For cytoplasmic and nuclear extracts, cells were first lysed in Buffer B (10 mM Hepes, pH 7.9, 10 mM KCl, 0.4% Nonidet P-40, 0.1 mM EDTA, 0.1 mM EGTA, 1 mM dithiothreitol, 1 mM phenylmethylsulfonyl fluoride) followed by centrifugation for 5 min at 4°C and 12,000 × g. The supernatant was cytoplasmic extract. The pellet (nucleus) was further lysed in Buffer C (20 mM Hepes, pH 7.9, 0.4 M NaCl, 0.1 mM EDTA, 1 mM dithiothreitol, 1 mM phenylmethylsulfonyl fluoride) [38]. The whole cell lysates, cytoplasmic extract and nuclear extract were used for immunoblotting assays as described previously [39].

### Luciferase gene reporter assays

RG3.6 cells treated with LiCl or NaCl were transfected with NF-AT firefly and TK renilla luciferase reporters. At 40 hrs post-transfection, luciferase activity was measured as we described before [40].

### Quantitative real time PCR

Total RNA was extracted from RG3.6 cells or primary neural precusor cells grown for 3 days in culture medium containing 3 mM LiCl or 3 mM control NaCl, and reverse transcribed into cDNA. Quantitative real time PCR was performed as described previously [41]. The primer pairs specific for the genes are indicated in Additional file 2.

### Affymetrix microarray analysis

Total RNA was extracted from RG3.6 cells grown for 3 days in culture medium containing 3 mM LiCl or 3 mM control NaCl (3 samples per condition). The integrity of the RNAs was examined using the Agilent platform (Agilent 2100 Bioanalyzer). Biotin-labeling of cDNA and subsequent hybridization using GeneChip^® ^Rat Genome 230 2.0 Array (Affymetrix) were carried out by the Transcriptional Facility Shared Resource of the Cancer Institute of New Jersey as described previously [42]. GeneSpring GX 9 software (Agilent) was used to screen genes whose RNA expression was significantly altered by lithium treatment.

### Statistical Analysis

Data were reported as mean ± standard deviation (SD). The ANOVA post hoc or Student's t test was used to assess significance of differences, and p values ≤ 0.05 and 0.01 were considered statistically significant and highly statistically significant, respectively [43].

## Abbreviations

ADP: adult rat dentate gyrus-derived neural precursor cells; BDNF: brain-derived neurotrophic factor; BrdU: bromodeoxyuridine; CNS: central nervous system; CNTF: ciliary neurotrophic factor; CRMP-2: collapsin response mediator protein-2; CsA: cyclosporin A; DAPI: 4', 6-Diamidino-2-phenylindole; GDNF: glial cell-derived neurotrophic factor; GSK-3β: glycogen synthase kinase 3β; IMPase: inositol monophosphate phosphatase; IPPase: inositol polyphosphate 1-phosphatase; LiCl: lithium chloride; LIF: leukemia inhibitory factor; NaCl: sodium chloride; NF-AT: nuclear factor of activated T-cells; NGFβ: nerve growth factor β; NGFγ: nerve growth factor γ; NT-3: Neurotrophin-3; Ppia: peptidylprolyl isomerase A; SMIT1: sodium-myo-inositol transporter 1.

## Competing interests

The authors declare that they have no competing interests.

## Authors' contributions

ZQ designed and performed the research, analyzed data and wrote the paper. DS and WY participated in the design and oversaw the research. All authors read and approved the final manuscript.

## Supplementary Material

Additional file 1 **Proliferation-related genes significantly up-regulated by lithium in RG3.6 cells**. RG3.6 cells were treated with 3 mM LiCl or 3 mM control NaCl (3 samples per condition), followed by RNA extraction and microarray analysis using the GeneChip^® ^Rat Genome 230 2.0 Array (Affymetrix) GeneSpring GX 9 software (Agilent) was used to screen genes whose RNA expression. Proliferation-related genes that were significantly up-regulated two folds by lithium are listed. Click here for file

Additional file 2 **Primers used for real-time PCR**. Primer sequences, gene names and their gene bank access numbers are listed. Click here for file
