# Supplementary material for: Lithium promotes neural precursor cell proliferation: evidence for the involvement of the non-canonical GSK-3β-NF-AT signaling
Source: Cell Biosci. 2011 May 3;1:18. doi: 10.1186/2045-3701-1-18 (PMC3125208; doi:10.1186/2045-3701-1-18)
Supplement: Additional file 1 — Proliferation-related genes significantly up-regulated by lithium in RG3.6 cells. RG3.6 cells were treated with 3 mM LiCl or 3 mM control NaCl (3 samples per condition), followed by RNA extraction and microarray analysis using the GeneChip® Rat Genome 230 2.0 Array (Affymetrix) GeneSpring GX 9 software (Agilent) was used to screen genes whose RNA expression. Proliferation-related genes that were significantly up-regulated two folds by lithium are listed. [file 2045-3701-1-18-S1.PDF]

Table 1. Proliferation-related genes significantly up-regulated more than two fold by lithium in RG3.6 cells.

Gene Symbol	Entrez Gene	GO Biological Process Term
Bub1b	171576	cell cycle /// cell division /// mitosis /// protein amino acid phosphorylation
Ccnb2	363088	cell cycle /// cell division
Cdca3 /// RGD1562067_predicted	297594 /// 501134	ubiquitin cycle /// cell cycle /// mitosis /// G1/S-specific positive regulation of cyclin-dependent protein kinase activity /// positive regulation of mitosis /// cell division
Ect2_predicted	361921	mitosis /// cytokinesis
Kif20a_predicted	361308	cytokinesis /// microtubule-based movement /// vesicle-mediated transport
Kif23_predicted	315740	Cytokinesis /// mitotic spindle elongation /// microtubule-based movement
Kifc1	294286	mitotic sister chromatid segregation /// microtubule-based movement /// cell cycle /// mitosis /// cell division
Mki67_predicted	291234	meiosis /// cell proliferation
Racgap1_predicted	315298	cytokinesis /// cytokinesis, contractile ring formation /// electron transport /// cytokinesis, initiation of separation /// intracellular signaling cascade /// spermatogenesis
Spbc25	295661	cell cycle /// mitosis /// cell division
Top2a	360243	DNA metabolic process /// DNA topological change /// DNA topological change /// DNA topological change /// chromosome segregation /// chromosome condensation /// embryonic cleavage /// positive regulation of transcription from RNA polymerase II promoter
Ttk_predicted	315852	protein amino acid phosphorylation /// mitotic spindle organization and biogenesis /// mitotic cell cycle spindle assembly checkpoint /// positive regulation of cell proliferation
Ube2c_predicted	296368	cytokinesis /// ubiquitin-dependent protein catabolic process /// cell cycle /// spindle organization and biogenesis /// mitosis /// cyclin catabolic process /// positive regulation of cell proliferation /// protein ubiquitination /// positive regulation of exit from mitosis /// phosphoinositide-mediated signaling
Uhrf1	316129	DNA repair /// transcription /// regulation of transcription, DNA-dependent /// protein modification process /// ubiquitin cycle /// transport /// response to DNA damage stimulus /// cell cycle

Table 1. Qu et al.
