# Supplementary material for: Lithium promotes neural precursor cell proliferation: evidence for the involvement of the non-canonical GSK-3β-NF-AT signaling
Source: Cell Biosci. 2011 May 3;1:18. doi: 10.1186/2045-3701-1-18 (PMC3125208; doi:10.1186/2045-3701-1-18)
Supplement: Additional file 2 — Primers used for real-time PCR. Primer sequences, gene names and their gene bank access numbers are listed. [file 2045-3701-1-18-S2.PDF]

Table 2. Primers used for real-time PCR

Gene	Forward primer	Reverse primer	Accession
CNTF	CCCATTTTGGTTTGCATTGG	GGATAGGTGGGCCATCCATT	NM_013166
GDNF	GGTCACCAGATAAACAAGCGG	GCCGGTTCCTCTCTCTTCG	NM_019139
LIF	TTGCCGTCTGTGCAACAAGT	ACACAGGGCACATCCACATG	NM_022196.2
NGFβ	GATCGGCGTACAGGCAGAAC	TCTCCCTCTGGGACATTGCT	XM_227525
NGFγ	TGCTCCTGCATGCCTGTTAC	CAGGGCGAGGAACAGGATC	NM_031523
NT3	GATATTTTGGCCGGAGGGAA	CCTCAAAAGGGCTGGGTTCT	NM_031073.2
Ppia	TCACCATCTCCGACTGTGGA	AAATGCCCGCAAGTCAAAGA	NM_017101

Table 2. Qu et al.
